# Severe hyperbilirubinemia prediction in neonates using a newly developed cord blood index (Çapa index): a promising tool: A retrospective cohort pilot study

**DOI:** 10.1097/MD.0000000000042516

**Published:** 2025-05-16

**Authors:** Mustafa Törehan Aslan, Zeynep İnce, Leyla Karadeniz Bilgin, Beril Yaşa, Meltem Bor, Asuman Çoban

**Affiliations:** a Division of Neonatology, Department of Pediatrics, Istanbul Faculty of Medicine, Istanbul University, Istanbul, Turkey; b Division of Neonatology, Department of Pediatrics, Koç University Hospital, Istanbul, Turkey.

**Keywords:** carboxyhemoglobin, hyperbilirubinemia, newborn jaundice, phototherapy, umbilical cord blood

## Abstract

Prevention of hyperbilirubinemia, among common reasons for outpatient visits and hospital readmissions during the neonatal period, depends on early diagnosis and effective treatment. Thus, discovering novel indices and parameters to predict severe hyperbilirubinemia is critical. The presence of hemolysis risk factors in newborns is not a prerequisite for treatment in most cases. We aimed to seek the role of a novel index (Çapa index), developed using umbilical cord blood carboxyhemoglobin (COHb) and total bilirubin levels, in predicting severe hyperbilirubinemia in the early neonatal period. In total, 290 term neonates were included in the present study, of which 171 were *direct antiglobulin test* positive with A, B, and O blood group system and/or rhesus factor incompatibility, and 119 were healthy controls without blood group incompatibility, sepsis, asphyxia, respiratory problems, pathologic weight loss, congenital anomaly, or need of intensive care. Çapa index was calculated by multiplying COHb (%) and total bilirubin (mg/dL) levels in umbilical cord blood and compared between the groups to predict its role in treatment requirements. COHb, bilirubin, and Çapa index were higher in the disease group than in healthy controls. In the disease group, cord blood bilirubin levels in the neonates needing phototherapy (PT) were unexpectedly significantly lower. In contrast, the COHb and Çapa index were higher than the ones without a need for treatment. In the disease group, the Çapa index had a significant predictive value in estimating PT requirements (area under the curve = 0.94). Standard hemolysis criteria have limited predictive value in the progression of hyperbilirubinemia. Çapa index, calculated as a practical biochemical index using umbilical cord blood COHb and bilirubin levels, can be a promising parameter in predicting severe hyperbilirubinemia and PT requirements in neonates.

## 1. Introduction

Hyperbilirubinemia is one of the most common reasons for clinic visits and hospitalization among newborns during the early neonatal period.^[1]^ The pooled incidence of severe neonatal hyperbilirubinemia was reported as 244/100,000 live births.^[2]^ The success of preventive measures against severe complications of indirect hyperbilirubinemia solely depends on early and effective therapeutic interventions. Clinically relevant jaundice develops mainly a few days after birth.^[3]^ The current medical procedure advising early discharge after delivery may delay diagnosis and treatment, increasing the risk of kernicterus-related sequelae and acute bilirubin encephalopathy.^[4]^ It was previously reported that 36% of the healthy term neonates developed severe hyperbilirubinemia and required rehospitalization in the USA.^[5]^ Given these risks, it is imperative to identify reliable and early predictors of severe hyperbilirubinemia.

There are still ongoing arguments regarding concerns about severe hyperbilirubinemia risk due to early discharge postdelivery. Thus, it urges clinicians to seek practical and predictive diagnostic novel parameters against severe neonatal hyperbilirubinemia.^[6]^ Relevant studies in the literature investigate the potential diagnostic value of umbilical cord blood bilirubin levels to establish a valid risk index for developing neonatal hyperbilirubinemia.^[5,7,8]^ However, more than just the analysis of bilirubin levels is needed to be an ideal predictor of the disease pathogenesis.^[9]^ In addition, the levels of unbound bilirubin, albumin, bilirubin-albumin binding capacity, and other biochemical parameters should be collectively analyzed to obtain a valid risk index for better clinical management of the disease.^[10]^ carboxyhemoglobin (COHb), a stable complex of CO and hemoglobin (Hb) molecules in erythrocytes, can become a promising diagnostic candidate. Mainly, COHb is endogenously produced after hem degradation during hemolysis and can be considered an early biomarker of the bilirubin formation process.^[11,12]^

The rationale behind the formulation of the novel index (Çapa index), which multiplies umbilical cord blood bilirubin (mg/dL) by COHb (%), lies in the complementary physiological information these parameters provide. Cord bilirubin reflects the net balance between fetal bilirubin production and placental clearance at birth, while COHb serves as a biomarker of endogenous carbon monoxide production – a direct indicator of heme catabolism and hemolytic activity.^[12,13]^ Elevated COHb levels have been correlated with increased hemolysis in neonates, especially in the context of blood group incompatibility.^[11]^ However, bilirubin levels in cord blood alone can be deceptively low at birth in infants with active hemolysis, due to efficient placental bilirubin excretion.^[10]^ By combining these 2 indicators through multiplication, the Çapa index amplifies risk prediction in infants where both ongoing hemolysis (high COHb) and bilirubin burden are relevant – even if bilirubin itself appears within normal limits at birth. This formulation therefore enhances early risk stratification for clinically significant postnatal hyperbilirubinemia and offers superior predictive sensitivity compared with each marker alone. The index is named in honor of the Istanbul Faculty of Medicine, which is colloquially known as “Çapa.” This is the institution where the research was conducted, and the index was developed. “We named the index “Çapa” as a nod to the Istanbul Faculty of Medicine (“Çapa”) where it was conceived. In the present study, we aimed to analyze whether the predictive role of a novel risk index for developing severe hyperbilirubinemia can be increased by considering both cord blood bilirubin and COHb levels.

## 2. Methods

### 2.1. Study population and design

Two hundred ninety healthy term newborns in total, needing no intensive care, were included in the study, of which 171 had *direct antiglobulin test (DAT*) positive A, B, and O blood group system (ABO) and/or rhesus factor (Rh) incompatibilities, and 119 were healthy controls without any blood incompatibility and phototherapy (PT) requirement. ABO incompatibility was defined as maternal blood type O and neonatal blood type A or B. Cases were considered ABO isoimmune hemolysis if the DAT of cord blood was positive; DAT-negative ABO mismatch cases (maternal O, infant A/B without hemolysis) were not included in the incompatibility group in this study. Similarly, Rh incompatibility was defined as maternal Rh-negative status with an Rh-positive neonate, and only those with a positive DAT indicating immune-mediated hemolysis were included. The exclusion criteria were as follows: having any postnatal adaptation issues, intensive care due to several disorders other than hyperbilirubinemia (respiration distress, sepsis, pathologic dehydration, etc.), and having congenital anomalies. No data on glucose-6-phosphate-dehydrogenase enzyme deficiency levels were available in infants in this cohort and therefore not analyzed. Decisions regarding PT requirements were made in accordance with the American Academy of Pediatrics clinical guidelines available during the study period. PT was initiated when total serum bilirubin levels exceeded hour-specific thresholds based on postnatal age, gestational age, and risk factors such as isoimmune hemolytic disease. None required exchange transfusion.

### 2.2. Sample size determination and sampling method

This study was designed as a retrospective cohort pilot study. The sample size was determined based on the total number of eligible term neonates who met the inclusion criteria and whose records were accessible during the study period between January 2016 and December 2021 at our center. No a priori power analysis was performed. All eligible neonates who fulfilled the predefined inclusion and exclusion criteria were included consecutively without randomization. Therefore, the sampling method was consecutive sampling.

### 2.3. Analysis of biochemical and clinical parameters

The following clinical and demographic parameters of the term neonates were recorded for further statistical analyses: gestational age, birth weight (g), blood group incompatibility type (ABO or Rh), DAT, umbilical cord blood total bilirubin (mg/dL), COHb (%), hematocrit (%) levels, and need for PT. Other than these parameters, the novel index, which we named as “Çapa index,” was calculated for each subject as follows:

Çapa index = umbilical cord blood bilirubin (mg/dL) × umbilical cord blood COHb (%).

### 2.4. Statistical analysis

All statistical analyses and comparisons were conducted using MedCalc package software (version 20.009; Ostend, Belgium). The data were represented as numbers, percentages, or median and 25 to 75th percentile values. The Gaussian distribution of continuous values was checked using the Kolmogorov–Smirnov test. Kruskal–Wallis test was used to compare multiple groups, and 2 individual groups were compared using the Bonferroni-corrected Mann–Whitney *U* test. Receiver operating characteristic (ROC) analysis was performed for Çapa index and cord blood bilirubin and COHb values, and the relevant cutoff, sensitivity, specificity, positive and negative likelihood ratios, and area under the curve (AUC) values were calculated. Statistical differences were considered significant at a threshold of *P* < .05.

### 2.5. Ethical statements and subjects

This study was approved by the Istanbul Faculty of Medicine Clinical Research Ethical Board (October 3, 2024/2920923) at Istanbul University and by the Helsinki Declaration. The data of term neonates born between January 2016 and December 2021 were acquired from the electronic database and patient files in the Istanbul Faculty of Medicine Hospital.

## 3. Results

This study included 290-term newborns. Of these, 171 had DAT-positive blood type (ABO or Rh) incompatibility, and 119 served as healthy, birth week- and birth weight-matched controls (Table 1). Novel Çapa index, umbilical cord blood total bilirubin, and COHb levels were significantly higher in newborns with blood type incompatibility than in the control group (*P* < .05). Umbilical cord blood Hb and hematocrit levels in the blood type-incompatible group significantly decreased compared with the control group (*P* < .05) (Table 1).

**Table 1:** Clinical characteristics and umbilical cord blood parameters of newborns with ABO and/or Rh incompatibility and control group.

	Newborns with ABO and/or Rh incompatibility (n = 171)	Control (n = 119)	*P* value
Gender, n (%)			
Female	54 (31.6)	58 (48.7)	.0034*
Male	117 (68.4)	61 (51.3)	
Birth weight (g)	3390 (3130–3600)	3430 (3220–3645)	.273
Gestational age (week)	38.6 (37.7–40)	39.1 (38.2–40)	.119
Birth type, n (%)			.210
Normal vaginal way	65 (38.0)	36 (30.3)	
Cesarean section	106 (62.0)	83 (69.7)	
Çapa index (novel)	1.8 (1.32–2.43)	1.04 (0.52–1.48)	<.0001*
Umbilical cord blood total bilirubin (mg/dL)	2.2 (1.8–2.9)	2 (1.4–2.5)	<.0001*
Umbilical cord blood COHb (%)	0.9 (0.6–1.2)	0.5 (0.3–0.8)	<.0001*
Umbilical cord blood Hb (g/dL)	15.9 (14–17.3)	16.3 (15.9–18.1)	<.0001*
Umbilical cord blood hematocrit (%)	47 (42.2–52)	49 (47–53)	.0003*

The data were presented as median (25–75 percentiles) or n (%).

ABO = A, B, and O blood groups, COHb = carboxyhemoglobin, Hb = hemoglobin, Rh = rhesus factor.

* *P* < .05 for statistical significance.

As summarized in Table 2, newborns with ABO and/or Rh blood type incompatibilities were subdivided into 2 groups depending on the PT requirement; of 171 neonates, 43% required PT(+) while 57% did not (PT[−]). The gestational age and birth weight values in the PT(+) group were significantly lower than the PT(−) and control groups (*P* < .05). Çapa index and COHb values were considerably higher in the PT(+) group compared with the ones in the PT(−) and control groups (*P* < .05). These 2 parameters were also higher in the PT(−) group compared with the control group (*P* < .05). Total bilirubin levels were significantly different between PT(+) and control groups (*P* < .05) and were higher in the PT(−) group compared with both the PT(+) and control groups (*P* < .05). Umbilical cord blood Hb and hematocrit levels in the PT(+) group significantly decreased compared with the control group (*P* < .05). Umbilical cord blood novel Çapa index and COHb were higher in the PT(+) group than in the PT(−) group. However, as a striking finding, contrary to expectations, the umbilical cord blood total bilirubin value was found to be lower, and the umbilical cord blood Hb and hematocrit values were higher in the PT(+) group than the ones in the PT(−) group.

**Table 2:** Clinical parameters and umbilical cord blood values of newborns with ABO and/or Rh incompatibility, who required phototherapy or not, and control group.

	Newborns with ABO and/or Rh incompatibility		Control (n = 119)	*P* value
	Phototherapy required (n = 73)	Phototherapy not required (n = 98)		
Gender, n (%)				
Female	27 (37.0)	27 (38.0)	58 (48.7)	.0058*
Male	46 (63.0)	71 (62.0)	61 (51.3)	
Birth weight (g)	3300 (2995–3495)	3490 (3240–3650)	3430 (3220–3645)	.001*
Gestational age (week)	38.3 (37.6–39.1)	39.3 (38–40.1)	39.1 (38.2–40)	.004*
Çapa index (novel)	2.42 (1.96–3)	1.44 (1.1–1.8)	1.04 (0.52–1.48)	<.0001*
Umbilical cord blood total bilirubin (mg/dL)	2 (1.68–2.4)	2.7 (1.8–3.1)	2 (1.4–2.5)	<.0001*
Umbilical cord blood COHb (%)	1.2 (1.1–1.4)	0.65 (0.4–0.9)	0.5 (0.3–0.8)	<.0001*
Umbilical cord blood Hb (g/dL)	16 (13.7–17.3)	15.5 (14–17.5)	16.3 (15.9–18.1)	<.0001*
Umbilical cord blood hematocrit (%)	48 (42–52)	46.5 (44–52)	49 (47–53)	<.0001*

The data were presented as median (25–75 percentiles) or n (%).

ABO = A, B, and O blood groups, COHb = carboxyhemoglobin, Hb = hemoglobin, Rh = rhesus factor.

* *P* < .05 for statistical significance.

**Figure 1.**
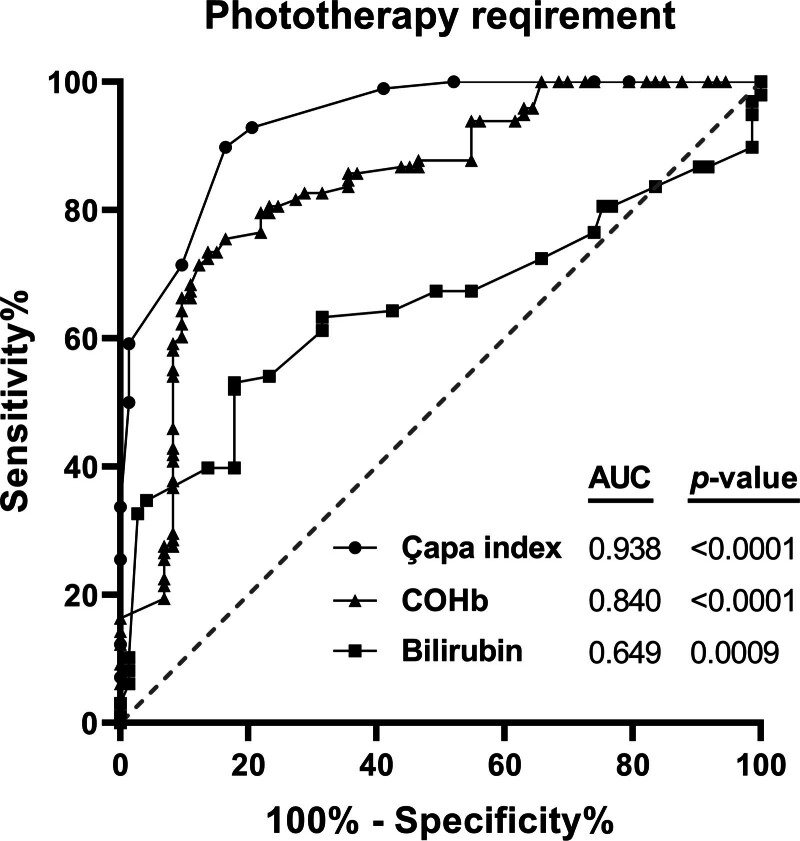
ROC analyses of the novel index, umbilical cord blood COHb, and total bilirubin levels in newborns with ABO and/or Rh incompatibility who required phototherapy (n = 73) and control group (n = 119). *P* < .05 for statistical significance. ABO = A, B, and O blood groups, AUC = area under the curve, COHb = carboxyhemoglobin, Hb = hemoglobin, Rh = rhesus factor, ROC = receiver operating curve.

ROC analysis was performed to analyze the predictive/diagnostic value of total bilirubin, COHb, and novel Çapa index in neonates with blood incompatibility for PT requirement, as depicted in Figure 1. All analyzed parameters showed a significant predictive value for PT requirement (Fig. 1, *P* < .05), while the novel Çapa index had the highest AUC value. In addition, in our subgroup analysis within the ABO incompatibility cohort, we compared infants with maternal O–neonatal A (O-A) and maternal O–neonatal B (O-B) blood type combinations. There was no statistically significant difference between the 2 groups in terms of PT requirement (*P* = .72) or median Çapa index values (*P* = .68).

## 4. Discussion

In this study, we evaluated the predictive value of a novel composite marker, the Çapa index, calculated as the product of cord blood bilirubin and COHb levels. We found that the Çapa index demonstrated significantly higher accuracy in predicting the need for PT compared with either marker alone. Notably, infants requiring PT had lower cord bilirubin but higher COHb and hematocrit levels, highlighting the clinical utility of combining these parameters to better identify at-risk neonates immediately after birth.

The current diagnostic tools can yield limited sensitivity in risk evaluations concerning assessing blood type incompatibilities and the need for PT.^[9]^ Therefore, there is a need for a more valid and practical noninvasive method in risk analysis of neonatal jaundice.^[6]^ Analyzing the umbilical cord blood parameters such as bilirubin, hematocrit, and COHb during delivery is low-cost, practical, and noninvasive.

In clinical practice, it is generally the attending neonatologist or pediatrician who is recommended to evaluate cord blood bilirubin and hematocrit levels, particularly in neonates identified as being at risk for hemolytic disease or hyperbilirubinemia. The American Academy of Pediatrics 2004 guidelines^[14]^ on the management of hyperbilirubinemia suggest that DAT should be performed on cord blood in at-risk infants (eg, those born to mothers with blood group O or Rh-negative status). Although routine measurement of cord bilirubin and hematocrit is not universally recommended for all neonates, in specific risk settings, clinicians may opt to obtain these measurements to facilitate early detection of hemolysis or anemia and guide timely management. Furthermore, clinical protocols in certain hospitals recommend obtaining these tests at birth in high-risk neonates based on local practices and risk stratification models.^[15]^ Performing such tests may also increase laboratory costs and manpower requirements. The evaluations and management of the patients included in our study (between 2016 and 2021) were based on these guidelines and local practices.

A retrospective study conducted with 1360 newborns stated that cord blood bilirubin levels showed a significant predictivity in neonatal hyperbilirubinemia (AUC = 0.80) within the first 48 hours of life.^[9]^ Sahan et al^[16]^ investigated the predictive value of cord blood bilirubin and bilirubin/albumin ratio in 217 healthy term newborns, and they suggested that these 2 parameters expressed significant value in estimating indirect neonatal hyperbilirubinemia along with the higher sensitivity and specificity of cord blood bilirubin/albumin ratio (74.2% and 61.8%, respectively). DAT positivity has been demonstrated to be a standard indicator among ABO incompatible births.^[17]^ However, DAT positivity alone does not always predict severe hyperbilirubinemia and does not have sufficient strength to indicate the need for PT.^[18,19]^ On the other hand, Tiras et al^[20]^ conducted a comparative clinical study exploring the predictive value of COHb over the DAT, and they concluded that cord blood COHb levels did not show a significant alteration and suggested the DAT as a more valid method in risk prediction of neonatal hyperbilirubinemia. Cord blood COHb levels were also found to be predictive for severe hyperbilirubinemia in ABO-incompatible and DAT-positive African-American newborns whose mothers have type O positive.^[21]^ We observed that COHb and total bilirubin levels in blood-incompatible jaundiced neonates were notably higher than in the control group. Similar to our findings, Lozar-Krivec et al^[11]^ stated that COHb levels were found to be markedly higher in hemolytic neonates with ABO incompatibility compared with healthy neonates. In another clinical study conducted with 616-term and near-term neonates, elevated total bilirubin levels of the umbilical cord were demonstrated as a valid risk factor for developing neonatal hyperbilirubinemia.^[8]^

Our subgroup analysis revealed no significant difference in PT requirement or Çapa index values between neonates with O-A and O-B blood group incompatibility. This suggests that the severity of hemolytic disease and the risk of developing clinically significant hyperbilirubinemia are comparable between these 2 ABO mismatch types. Al-Omran et al^[22]^ stated, “positive DAT was more prevalent in the O-B than the O-A incompatibility (43.5% vs 29.2%, *P* < .001). A greater odds of PT need were observed in the O-B versus O-A incompatibility across various strata. Readmission for neonatal indirect hyperbilirubinemia, use of 360° exposure PT, or intravenous immunoglobulin administration was more prevalent in the O-B than the O-A incompatibility (13.2% vs 5.0%, *P* < .001). These findings differ from the results reported by Al-Omran et al, who found a statistically significant difference in the need for PT between O-A and O-B incompatible term neonates in their retrospective cohort study. This difference in results may also be related to the number and diversity of patients. Together with all these observations, it may suggest that both O-A and O-B incompatibility should be carefully considered in terms of DAT positivity, jaundice monitoring, and early intervention. Jones et al^[5]^ conducted another cord blood bilirubin-based risk assessment strategy in 1411-term neonates. They found bilirubin levels as a robust predictive risk factor for jaundice in newborns whose mothers have O blood type. Notably, our results revealed a striking finding: contrary to expectations, the total bilirubin value in umbilical cord blood was lower in the group requiring PT than in the group not. In addition, the Hb and hematocrit values of umbilical cord blood were higher in the group requiring PT than in the group not requiring it. This seemingly paradoxical finding – lower cord blood bilirubin but higher Hb and hematocrit in neonates who later required PT. The observed paradox, wherein neonates requiring PT exhibit lower umbilical cord blood bilirubin levels yet higher Hb and hematocrit values, can be elucidated through the interplay of fetal physiology and placental function. During fetal life, unconjugated bilirubin produced from red blood cell turnover is transferred across the placenta to the maternal circulation for elimination, effectively maintaining low fetal bilirubin levels despite ongoing hemolysis. Concurrently, fetal hypoxia stimulates erythropoietin production, leading to increased erythropoiesis and elevated Hb and hematocrit levels – a compensatory mechanism to enhance oxygen delivery. Postnatally, the cessation of placental bilirubin clearance, combined with the breakdown of a larger red blood cell mass, results in a rapid rise in serum bilirubin levels, necessitating PT. This sequence of events reconciles the initial low bilirubin levels with subsequent hyperbilirubinemia in these neonates.^[23–26]^

In parallel to our findings, Varal et al^[27]^ reported in a study conducted with 221 preterm infants that COHb levels expressed a strong validity (AUC = 0.95) in predicting PT requirements. Previous studies reported that the involvement of additional parameters besides bilirubin level, such as albumin level^[28]^ or DAT^[29]^ in cord blood, could provide better predictivity for neonatal hyperbilirubinemia. Inconsistent cutoff values of cord blood bilirubin measured in previous studies in the decision of PT ^[30]^ or early postnatal discharge^[31]^ urge clinicians to seek supportive parameters. In the present study, we developed a novel risk index called the “Çapa index” by multiplication of cord blood COHb and bilirubin levels in term neonates to increase the sensitivity of prediction of PT requirement in blood-incompatible newborns. We found that the Çapa index was significantly higher in blood-incompatible neonates compared with the control group. We also found that the novel Çapa index (AUC = 0.94) had a better predictive value compared to individual cord blood COHb (AUC = 0.84) and bilirubin (AUC = 0.65) levels in PT requirement based on the ROC analyses. We received a valuable reviewer comment and suggestion during the peer review process of the article to potentially highlight cases with high hemolysis and low bilirubin levels, which was another alternative formulation that could come up with the COHb/bilirubin ratio (perhaps under the title of Çapa index-2, which could be useful for further study and should be evaluated with interest). We acknowledge that this alternative index was suggested to us by the relevant reviewer who evaluated our article. However, while evaluating this approach, we preferred to retain the product-based Çapa index as it provides a more intuitive reflection of the combined risk from both high hemolysis and bilirubin load. Furthermore, the Çapa index showed superior predictive performance (AUC = 0.94) in our cohort. Therefore, the product formulation was preferred due to its clinical applicability and sufficient predictive power. In this manner, we reached a higher sensitivity with the Çapa index in the prediction of PT requirement rather than in the hemolysis indication, suggesting that the clinicians should closely follow up the neonates with a higher Çapa index value (cutoff > 1.76).

### 4.1. Limitations and future direction

We presented a unique indicator called the “Çapa index,” which is a better predictive risk parameter in the PT requirement of neonates with blood incompatibility. The present study has certain limitations. First, the study population is relatively small (171 blood incompatible and 119 healthy neonates). Second, our study comprises single-center data. By increasing the cohort size and the number of study centers, more comprehensive outcomes can be acquired for more reliable interpretations of the novel Çapa index’s predictive value in estimating the need for PT in early neonatal hyperbilirubinemia. This study has notable strengths, including the use of objective, routinely collected cord blood parameters and standardized PT criteria applied across all subjects. However, in terms of internal validity, its retrospective nature may introduce selection bias and unmeasured confounding, such as undetected glucose-6-phosphate-dehydrogenase deficiency. The inclusion of only DAT-positive infants may also limit the variability of hemolytic severity within the cohort. Regarding external validity, this single-center study, limited to term neonates with isoimmune hemolysis, may not be generalizable to preterm infants, DAT-negative cases, or populations with different genetic or clinical characteristics. With validation from future multicenter prospective studies, the newly introduced “Çapa index’’ may emerge as a strong predictive tool for severe hyperbilirubinemia and holds potential for inclusion in clinical practice guidelines.

## 5. Conclusion

Standard criteria for hemolysis have limited power in estimating early hyperbilirubinemia progression in term newborns. Çapa index we designed as a practical biochemical risk indicator by taking into account both umbilical cord blood COHb and bilirubin levels has superior sensitivity over cord blood bilirubin and COHb levels alone and can be a promising noninvasive risk predictor in early neonatal hyperbilirubinemia and PT requirement.

## Author contributions

**Conceptualization:** Mustafa Törehan Aslan, Zeynep İnce, Leyla Karadeniz Bilgin, Asuman Çoban.

**Data curation:** Mustafa Törehan Aslan, Beril Yaşa, Meltem Bor.

**Formal analysis:** Mustafa Törehan Aslan, Zeynep İnce, Leyla Karadeniz Bilgin, Asuman Çoban.

**Investigation:** Mustafa Törehan Aslan, Beril Yaşa, Meltem Bor.

**Writing – original draft:** Mustafa Törehan Aslan, Zeynep İnce, Leyla Karadeniz Bilgin, Asuman Çoban.

**Writing – review & editing:** Mustafa Törehan Aslan, Zeynep İnce, Leyla Karadeniz Bilgin, Asuman Çoban.
